# Gender disparity in quality of life in patients with atrial fibrillation during the Syrian conflict: An observational cohort study

**DOI:** 10.1016/j.hroo.2024.12.010

**Published:** 2025-01-09

**Authors:** Ibrahim Antoun, Alkassem Alkhayer, Aref Jalal Eldin, Alamer Alkhayer, Khaled Yazji, Riyaz Somani, G. André Ng, Mustafa Zakkar

**Affiliations:** 1 Department of Cardiovascular Sciences, University of Leicester, Leicester, United Kingdom; 2 Department of Medicine, University of Aleppo, Aleppo, Syria; 3 Department of Cardiology, University of Tishreen’s Hospital, Latakia, Syria; 4 Department of Cardiology, The View Hospital, Qatar, Qatar; 5 Department of Cardiology, University Hospitals of Leicester NHS Trust, Glenfield Hospital, Leicester, United Kingdom; 6 Department of Research, NIHR Leicester Biomedical Research Centre, Leicester, United Kingdom; 7 Department of Cardiac Surgery, University Hospitals of Leicester NHS Trust, Glenfield Hospital, Leicester, United Kingdom; 8 Department of Medicine, University of Damascus, Damascus, Syria

**Keywords:** Atrial fibrillation, Quality of life, Gender, Syria, Conflict

## Abstract

**Background:**

The EuroQoL 5 dimensions is the first validated questionnaire to assess quality of life (QoL) in patients with atrial fibrillation (AF) in Syria.

**Objective:**

This study aimed to evaluate the gender disparity in QoL in Syrian patients with AF during the ongoing conflict.

**Methods:**

The study involved patients admitted to the emergency department of Tishreen University Hospital in Latakia, Syria, with AF as the primary diagnosis between June 2023 and June 2024. Arabic versions of the EuroQoL 5 dimensions were administered to patients on admission, and their demographic data were taken from their medical notes.

**Results:**

A total of 406 satisfied the study criteria, of whom 180 (44%) were males; and the median age was 67 (57- 73) years. Compared with men, women had more congestive cardiac failure (CCF) (32% vs 22%; *P*=.001) and were more uneducated (48% vs 27%; *P*<.001). Females demonstrated poorer QoL across all scoring dimensions (activities: 2.4 vs 1.7; mobility: 2.5 vs 1.6; self-care: 2.6 vs 1.8; pain: 2.6 vs 1.7; anxiety: 2.9 vs 1.8; EuroQol Visual Analogue Scale: 49.8 vs 79.8), all of which had *P*<.001. Multivariable logistic regression demonstrated that women (odds ratio [OR]: 5.2; 95% confidence interval [CI] 2.2–7.6; *P*<.001 and OR 6.2; 95% CI 3.1–9.7; *P*<.001) and CCF (OR 3.3; 95% CI 1.5–6.9; *P*<.001 and OR 4.2; 95% CI 2.2–7.3; *P*<.001) were independently associated with poor QoL.

**Conclusion:**

Syrian women admitted with AF had poorer QoL than did their men counterparts. CCF and female sex were independent predictors of poor QoL.

**Key Findings**

▪ This study is the first to explore gender differences in QoL among patients with AF in a conflict zone, offering insights into health care challenges in resource-limited settings.
▪ Female patients with atrial fibrillation in Syria reported significantly poorer quality of life than did male patients.
▪ Congestive cardiac failure and female gender were identified as independent predictors of poor quality of life.

## Introduction

Health-related quality of life (QoL) is self-perceived well-being related to diseases or treatments.^1^ Studies on Syrian QoL are mainly conducted on Syrian refugees abroad.

Atrial fibrillation (AF) is the most common sustained arrhythmia, and its prevalence in low- to middle-income countries is underestimated.^2^ Although AF in the Western world is extensively studied, little data corroborate AF demographics and management patterns in the Middle East, with 4 epidemiological data registries.^3^ Of the total AF research, 0.7% was Middle Eastern–based research.^4^ AF is known to impair QoL, as assessed by various studies.^5^ However, in conflict countries, this is less documented. One of these countries, Syria has been in conflict since 2011 and deprived of health care funding and resources, particularly exacerbated during the coronavirus disease 2019 pandemic and cholera outbreak.^6^^,^^7^ Less than half of its hospitals operate at total capacity, and >50% of its health care workforce is forced to leave because of conflict. More than half the Syrian population suffer from poverty, which is suggested to increase AF risk.^8^^,^^9^

A recent effort has been made to explore AF management outcomes in Syria.^10^, ^11^, ^12^ However, the QoL of patients with AF in Syria is a critical concern, given the country's damaged health care.^13^^,^^14^ The EuroQoL 5 dimnesions (EQ-5D) has recently validated the QoL assessment in patients with AF in Syria.^15^ It is a standardized generic instrument to assess health and QoL outcomes. It is available and validated in most significant languages with cultural adaptations. It provides index values and a simple descriptive profile of health status.^16^ In AF-based studies in the developed world, females tend to report more symptoms, more significant physical limitations, and increased emotional stress than do males, likely because of biological and sociocultural factors.^17^ In conflict zones, these disparities are likely amplified by disrupted access to care, increased caregiving burdens, and societal expectations, leaving females particularly vulnerable to diminished health outcomes and well-being. *Recognizing this gender impact is fundamental for creating targeted interventions that effectively address the distinct needs of male and female patients in conflict-affected areas*. This study assesses the gender disparity in QoL in patients with AF admitted to a tertiary center in Latakia, Syria.

## Methods

### Population and data collection

This single-center retrospective observational cohort study was conducted at Tishreen University Hospital, Latakia, Syria, between June 2023 and June 2024. The hospital is a large government-operated public institution associated with Tishreen University, and it serves as the tertiary health care center for the city and the surrounding areas. The hospital has ∼860 beds and provides free health care. The hospital sees ∼50,000–60,000 inpatients annually and more outpatients seeking care in several medical departments. The study included consecutive Arabic-speaking patients older than 18 years without an apparent cognitive deficit admitted through the emergency department and treated for AF as the main diagnosis. Patients with secondary AF and patients in intensive care or high-dependency units were excluded. Medical students who administered the questionnaires were in their fifth year of medical education and had prior clinical experience through rotations in various medical disciplines. They underwent formal training tailored to administering the EQ-5D and EuroQol Visual Analogue Scale (EQ-VAS) instruments. This training included detailed sessions on the structure and purpose of the questionnaires, ethical considerations during data collection, and techniques to ensure standardized administration. The training also incorporated mock interviews with standardized patients, followed by feedback sessions to enhance reliability. Supervision by senior clinicians and researchers was provided throughout the process to ensure consistency in data collection and minimize variability. The questionnaires are administered upon admission. Medical notes and records were examined to determine the medical history and treatments provided.

The study was written according to strengthening the reporting of observational studies in epidemiology guidelines.^7^ The research reported in this article adhered to the Declaration of Helsinki. The project was conducted as part of an audit approved by the hospital board and involved the prospective analysis of retrospectively collected anonymized data. Therefore, the hospital board waived the need for consent.

### EQ-5D and EQ-VAS questionnaires

The EQ-5D is a preference-based instrument used to assess QoL. Although translated into multiple languages, it has been translated and culturally adapted into Arabic in several countries, including Jordan and Saudi Arabia.^8^^,^^9^ The EQ-5D includes a classification system of 5 dimensions—mobility, usual activities, self-care, anxiety/depression, and pain/discomfort. Each dimension is coded either as 1 (no problem), 2 (some problem), or 3 (severe problem).^3^ The EQ-VAS is a straight line ranging from 0 (representing the worst imaginable health) to 100 (representing the best imaginable health), allowing individuals to define their current health status. They were both validated in Syrian patients with AF.^15^

### Study outcomes

The primary outcome is the disparity in QoL between male and female genders. Secondary outcomes include predictors of poor QoL in these patients.

### Statistical analysis

Continuous variables were expressed as median and interquartile range. Categorical variables were expressed as count and percentage. The Pearson χ^2^ or Fisher exact test was used to compare categorical variables between groups. Student *t* tests and Kruskal-Wallis tests were used to compare continuous variables between the groups, depending on the normality of the distribution. Two logistic regression models were used to assess predictors of poor QoL measured by the sum of the scores of the 5 dimensions of the EQ-5D and EQ-VAS. Satisfactory QoL in this study was defined by EQ-5D score ≤ 7 and EQ-VAS score > 50. A significance level of *P* < .05 was set. Statistical analysis was performed using Prism version 10.0 for Mac (GraphPad Software, San Diego, CA).

## Results

### Patient characteristics

A total of 406 satisfied the study criteria, of whom 180 were men (44%). All of them were Syrian and spoke and understood Arabic. Demographic characteristics are summarized in Table 1. The median age was 67 years, and the most common comorbidity was hypertension present in 173 patients (43%). Compared with men, women had more congestive cardiac failure (CCF) (32% vs 22%; *P*=.001) and were more uneducated (48% vs 27%; *P*<.001). A higher proportion of males completed primary/secondary school than females (57% vs 44%; *P*<.001).

**Table 1:** Demographic characteristics of the study participants

Characteristic	Overall (N=406)	Male (n=180)	Female (n=226)	*P*
Cardiovascular risk factors				
Age (y)	67 (56–73)	67 (56–75)	65 (57–71)	.22
Known AF	92 (23)	54 (24)	38 (21)	.74
Hypertension	173 (43)	79 (44)	94 (42)	.84
Ischemic heart disease	94 (23)	48 (27)	46 (20)	.32
Diabetes mellitus	122 (30)	50 (28)	72 (32)	.27
Cerebrovascular event	132 (19)	60 (19)	72 (21)	.33
Congestive heart failure	112 (28)	39 (22)	73 (32)	.001
CHA_2_DS_2_-Vasc score	2 (1–3)	2 (1–3)	2 (1–3)	.63
Highest education completed				
Uneducated	157 (39)	49 (27)	108 (48)	<.001
Primary/secondary school	201 (50)	102 (57)	99 (44)	<.001
University	48 (12)	29 (16)	19 (8)	.07
Other comorbidities				
Anemia	111 (18)	31 (17)	41 (18)	.18
Thyroid disease	19 (5)	10 (6)	6 (3)	.1
Dementia	46 (11)	12 (21)	15 (7)	.56
Malignant neoplasm	17 (4)	19 (11)	7 (3)	.85
Chronic liver failure	23 (6)	10 (6)	13 (6)	.35
Chronic lung disease	36 (9)	13 (7)	7 (3)	.71
AF treatment				
Rhythm control	156 (38)	92 (41)	64 (36)	.21
Rate control	250 (62)	159 (64)	173 (59)	.13

Values are presented as median (interquartile range) or n (%).

AF = atrial fibrillation.

### Outcomes

**Figure 1:**
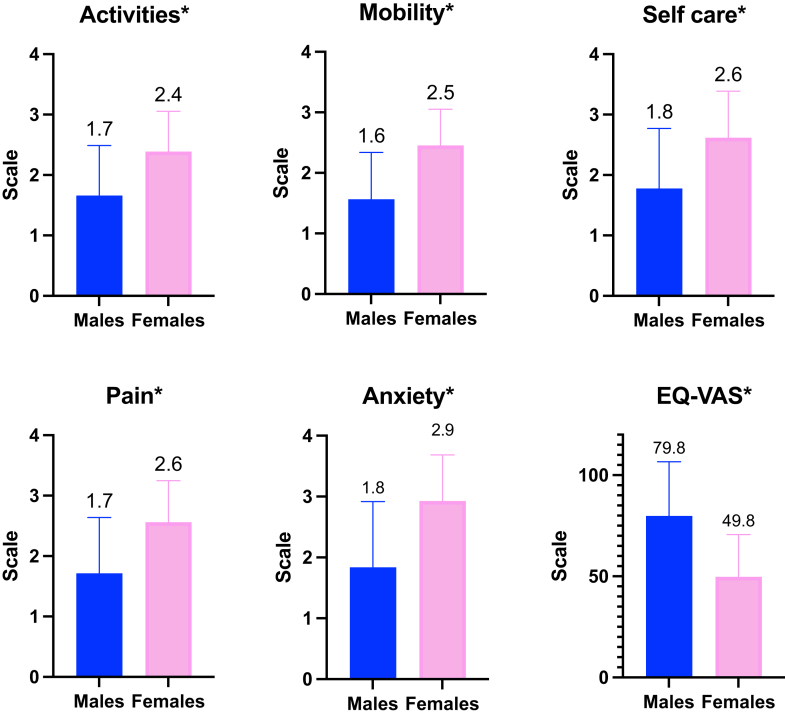
Gender-stratified quality of life outcomes in our study. Higher numbers in activities, mobility, self-care, pain, and anxiety indicate poorer quality of life, while higher numbers in EuroQol Visual Analogue Scale (EQ-VAS) indicate better quality of life. ∗ *P*<.001.

Figure 1 compares EQ-5D and EQ-VAS between males and females. Females demonstrated poorer QoL across all scoring dimensions (activities: 2.4 vs 1.7; mobility: 2.5 vs 1.6; self-care: 2.6 vs 1.8; pain: 2.6 vs 1.7; anxiety: 2.9 vs 1.8; EQ-VAS: 49.8 vs 79.8), all of which had *P*<.001.

Logistic regression analysis is presented in Table 2. The univariable analysis showed that females (odds ratio [OR] 4.3; 95% confidence interval [CI] 3.6–5; *P*<.001), CCF (OR 2.3; 95% CI 1.3–5.4; *P*=.03), and lack of education (OR 1.8; 95% CI 1.1–3.5; *P*=.04) were associated with lower EQ-5D scores. Similarly, univariable analysis demonstrated that females (OR 5.5; 95% CI 2.1–8.4; *P*<.001) and CCF (OR 3.5; 95% CI 2–5.4; *P*=.03) were associated with lower EQ-VAS scores. Multivariable analysis demonstrated that females (OR 5.2; 95% CI 2.2–7.6; *P*<.001 and OR 6.2; 95% CI 3.1–9.7; *P*<.001) and CCF (OR 3.3; 95% CI 1.5–6.9; *P*<.001 and OR 4.2; 95% CI 2.2–7.3; *P*<.001) were independently associated with lower EQ-5D and EQ-VAS scores, respectively.

**Table 2:** Logistic regression analysis to assess predictors of poor quality of life

Variable	EQ-5D				EQ-VAS			
	Univariable analysis		Multivariable analysis		Univariable analysis		Multivariable analysis	
	OR (95% CI)	*P*	OR (95% CI)	*P*	OR (95% CI)	*P*	OR (95% CI)	*P*
Females (vs males)	4.3 (3.6–5)	<.001	5.2 (2.2–7.6)	<.001	5.5 (2.1–8.4)	<.001	6.2 (2.9–9.7)	<.001
Congestive heart failure	2.3 (1.3–5.4)	.03	3.3 (1.5–6.9)	<.001	3.5 (2.2–6.6)	.01	4.2 (2.2–7.3)	<.001
Uneducated vs (school and university education)	1.8 (1.1–3.5)	.04	1.4 (0.9–2.1)	.07	1.5 (0.9–2.1)	.06		
Age (for every 10-y increase)	1.2 (0.7–1.8)	.65			1.1 (0.5–2.2)	.65		
Ischemic heart disease (yes vs no)	1.2 (0.3–2.5)	.23			1.3 (0.5–2.3)	.23		
Diabetes mellitus (yes vs no)	1.1 (0.6–2.7)	.28			1.2 (0.8–1.9)	.28		
Cerebrovascular event (yes vs no)	1.3 (0.3–2.3)	.10			1.1 (0.5–2.3)	.10		
AF history (yes vs no)	1.1 (0.4–2.2)	.89			1 (0.9–1)	.89		
CHA_2_DS_2_-Vasc score (per point increase)	1.2 (0.5–1.8)	.69			1.3 (0.6–2.1)	.10		
Hypertension (yes vs no)	0.9 (0.5–1.8)	.25			1 (0.9–1)	.11		
Anemia (yes vs no)	1 (0.9–1)	.91			0.8 (0.5–1.5)	.74		
Thyroid disease (yes vs no)	1 (0.9–1)	.94			1 (0.9–1)	.84		
Dementia (yes vs no)	1 (0.9–1)	.21			0.9 (0.7–1.3)	.21		
Active malignancy (yes vs no)	0.8 (0.6–1.6)	.87			1 (0.9–1)	.87		
Chronic liver failure (yes vs no)	1 (0.9–1)	.51			0.8 (0.5–1.9)	.51		
Chronic lung disease (yes vs no)	1.1 (0.9–1.3)	.76			1 (0.9–1)	.89		
Rhythm control (vs rate control)	1.1 (0.8–1.4)	.33			0.7 (0.2–2.3)	.33		

AF = atrial fibrillation; CI = confidence interval; OR = odds ratio.

## Discussion

This is the first study to assess gender disparity in QoL in patients with AF in a conflict-stricken country. This study reports 2 main findings that are unique to conflict-stricken Syria. First, females admitted with AF had worse QoL than did males, evidenced by higher EQ-5D scores across all dimensions and lower EQ-VAS scores. Second, CCF and female sex were poor predictors of poor QoL. Using the EQ-5D questionnaire in our study is noteworthy, as it is a validated tool for assessing QoL across various populations, including those with cardiovascular conditions, including AF.^18^ In a previous cohort, we recently validated the EQ-5D tool in assessing patients with AF in Syria, and it remains the only validated questionnaire for evaluating QoL in Syrian patients.^15^ CCF and lack of education were more prevalent in females in our cohort. This finding underscores the intersection of gender, education, and health outcomes, suggesting that educational disparities may contribute to the observed differences in QoL. Previous studies have indicated that lower educational attainment often correlates with poorer health literacy, adversely affecting self-management and treatment adherence in chronic conditions such as AF.^19^^,^^20^ Also, CCF prevalence in females in this study may exacerbate their QoL issues, as CCF is known to significantly impair functional status and overall well-being, according to previous studies.^21^^,^^22^ The interaction between QoL and CCF has been well-documented in different populations, indicating that the burden of heart failure could disproportionately affect females, especially in settings where health care resources are limited.^23^^,^^24^

The multivariable logistic regression analysis confirms that females and CCF are independent predictors of poor QoL. Worse QoL in women aligns with existing literature from the developed world, indicating that females with AF reported worse QoL than males.^25^ There are no data regarding these disparities in conflict-stricken countries. This disparity is justified by several factors, including biological, psychological, and social.^25^^,^^26^ The unique sociopolitical and cultural context may further exacerbate these disparities in the Syrian cohort. Psychosocial stressors, such as caregiving responsibilities, societal expectations, and restricted autonomy, disproportionately burden females in conflict-stricken zones. These stressors could lead to heightened anxiety and depression, as evidenced by higher anxiety/depression scores among the female gender in our study. A previous Syrian study demonstrated that the female gender had increased levels of physical impairment and depression.^27^ Another study showed that Syrian females had less marital satisfaction, life satisfaction, and self-esteem than did males.^28^ In addition, systemic inequities such as lower levels of education and limited access to health care disproportionately affect women, hindering their ability to seek timely care or understand and adhere to complex treatment regimens.

Moreover, the unique sociopolitical context of Syria, marked by ongoing conflict, further aggravates this gender disparity observed in our study. Females in conflict zones face additional stressors that could negatively affect their overall QoL and health outcomes. The intersection of gender, education, and health in such environments highlights the urgent need for targeted interventions that address the specific challenges faced by women with AF.^29^

Addressing these disparities requires a multifaceted approach. Syrian doctors should be trained to recognize gender-specific symptoms and barriers to care in AF management. Tailored treatment protocols and patient education strategies could mitigate these disparities. For instance, incorporating mental health support and patient-centered counseling for women with AF could address the psychological and emotional burden. Increasing health literacy among women through community programs and educational campaigns could empower them to recognize symptoms, seek care promptly, and adhere to prescribed treatments. Policymakers should prioritize improving health care access for women, particularly in conflict-stricken areas, by implementing mobile health units, subsidizing treatments, and offering mental health resources. Integrating gender-specific health policies into broader health care reforms can also address systemic inequities.

These findings have profound implications, suggesting that health care providers must consider gender-specific factors when developing treatment plans for patients with AF, particularly in conflict-affected regions where access to care may be limited.^24^^,^^30^

The findings of this study highlight the need for further research on gender disparities in AF and other chronic conditions in conflict-stricken zones. Future studies should examine the roles of trauma and chronic stress as mediators of AF symptoms and QoL outcomes in populations affected by conflict. It is also important to assess the effectiveness of gender-sensitive health care interventions, such as tailored education programs and support groups, in improving AF outcomes in women. Conducting multicenter studies across conflict-affected regions could help evaluate how cultural, systemic, and environmental factors influence gender disparities in chronic disease outcomes. By addressing these research gaps and implementing evidence-based policies, health care systems in conflict-stricken zones can become more inclusive and better meet the needs of vulnerable populations.

### Limitations

Data collection was limited to a single tertiary care center displacement in Latakia. Given the significant heterogeneity in the level and quality of hospital staffing and supplies, our results might not be generalizable to other hospitals nationwide. In addition, our analysis included only routinely collected data within the medical records and by the number of patients who presented to the center. Therefore, other variables potentially affecting QoL may have yet to be identified. While common in health research, the dependence on self-reported measures for QoL can introduce bias. Patients may under- or overreport their QoL because of several factors, including a lack of awareness about their health status or social desirability bias. This limitation is particularly relevant in the context of a conflict-affected region such as Syria, where stress and trauma may impact patients' perceptions of their health. There was difficulty distinguishing primary AF from secondary AF because of the lack of comprehensive diagnostic data, such as echocardiographic findings or detailed biochemical evaluation. As patient history was obtained solely through interviews and no electronic health records were available, it was not possible to confirm whether conditions such as severe valvular disease, advanced heart failure, or thyroid dysfunction were causative factors for AF. This may have introduced some heterogeneity in the studied cohort. Another limitation of this study is the reliance on self-reported measures for assessing QoL, which presents the possibility of self-reporting bias. Patients may under- or overreport their QoL because of various factors, including a lack of awareness about their health status, psychological influences such as stress or trauma, or a desire to present themselves in a certain way (social desirability bias). This limitation is particularly relevant in the context of a conflict-stricken region such as Syria, where prolonged exposure to stress and limited access to health care may influence patients' perceptions of their health and well-being, potentially skewing the results in either direction.

## Conclusion

Females and patients with CCF were independently associated with worse QoL during the Syrian conflict. This study contributes to understanding gender disparities in QoL among patients with AF in Syria. It highlights the need for comprehensive health care strategies that address the unique challenges faced by female patients, particularly those with CCF. Future research should focus on developing targeted interventions that can enhance QoL for these patients, considering the multifaceted factors influencing their health outcomes.
